# Global connectivity in genome-scale metabolic networks revealed by comprehensive FBA-based pathway analysis

**DOI:** 10.1186/s12866-021-02357-1

**Published:** 2021-10-25

**Authors:** Yajie Gao, Qianqian Yuan, Zhitao Mao, Hao Liu, Hongwu Ma

**Affiliations:** 1 Biodesign Center, Key Laboratory of Systems Microbial Biotechnology, Tianjin Institute of Industrial Biotechnology, Chinese Academy of Sciences, Tianjin, China; 2 College of Biotechnology, Tianjin University of Science & Technology, Tianjin, China

**Keywords:** Genome-scale metabolic network, Flux balance analysis, Bow-tie structure, Giant strongly connected component (GSC), Network connectivity, Pathway analysis

## Abstract

**Background:**

Graph-based analysis (GBA) of genome-scale metabolic networks has revealed system-level structures such as the bow-tie connectivity that describes the overall mass flow in a network. However, many pathways obtained by GBA are biologically impossible, making it difficult to study how the global structures affect the biological functions of a network. New method that can calculate the biologically relevant pathways is desirable for structural analysis of metabolic networks.

**Results:**

Here, we present a new method to determine the bow-tie connectivity structure by calculating possible pathways between any pairs of metabolites in the metabolic network using a flux balance analysis (FBA) approach to ensure that the obtained pathways are biologically relevant. We tested this method with 15 selected high-quality genome-scale metabolic models from BiGG database. The results confirmed the key roles of central metabolites in network connectivity, locating in the core part of the bow-tie structure, the giant strongly connected component (GSC). However, the sizes of GSCs revealed by GBA are significantly larger than those by FBA approach. A great number of metabolites in the GSC from GBA actually cannot be produced from or converted to other metabolites through a mass balanced pathway and thus should not be in GSC but in other subsets of the bow-tie structure. In contrast, the bow-tie structural classification of metabolites obtained by FBA is more biologically relevant and suitable for the study of the structure-function relationships of genome scale metabolic networks.

**Conclusions:**

The FBA based pathway calculation improve the biologically relevant classification of metabolites in the bow-tie connectivity structure of the metabolic network, taking us one step further toward understanding how such system-level structures impact the biological functions of an organism.

**Supplementary Information:**

The online version contains supplementary material available at 10.1186/s12866-021-02357-1.

## Background

Metabolism is the basic process of living organisms which provides building blocks and energy for cellular growth. The availability of genome sequences for a great number of organisms enables the reconstruction of genome-scale metabolic networks (GSMNs) containing thousands of enzyme-catalyzed reactions and metabolites [1, 2]. Understanding the evolution of the complex network structure and its relationship with the biological functions has been key scientific questions in the last decades. Two different approaches have been widely used to study the structure and function of GSMNs, the graph based analysis (GBA) [3] and flux balance analysis (FBA) [4]. The GBA approach is useful for the discovery of several system-level structural features of complex GSMNs, such as scale-free, modularity, and bow-tie connectivity [5–7]. These features are often common to different types of complex networks and are hypothesized to be related with the robustness and adaptive evolution of complex systems [8–12]. However, it is not straightforward to apply these general network-level organization principles to guide the understanding and design of real biological functions. There is still a big gap between network biology and understanding which reactions should be targeted in a metabolic network to maximize the flux toward an objective product [13]. By contrast, the FBA approach is more suitable for biological function analysis and has been widely applied to simulate growth rates, predict optimal pathways from a substrate to a product, and identify target reactions for increased production of desired biochemicals [14, 15]. In FBA approach, the stoichiometric relationships between reactions in a network are used as constraints to determine the solution space of the steady-state fluxes. One main difference between the two approaches is that FBA tends to calculate the optimal pathways (e.g. maximal product rate at an assigned substrate uptake rate) between a substrate and a product (or biomass) in a specific metabolic network, while GBA comprehensively calculates the pathways between all pairs of metabolites in a network to identify the system-level organizational structure. However, many pathways in a metabolic network calculated using GBA are not biologically feasible due to a simplified representation of the metabolic network [16, 17]. Advanced methods such as atom mapping were proposed to address this problem [18, 19], but these methods either require accurate atom mapping information of all reactions in a network, which is often not available in published GSMNs [16, 20], or require solving complex mixed-integer linear optimization problems to exclude the impossible pathways [17]. In addition, these methods were often applied to calculate pathways between a small number of substrates and products but have not been used to analyze the connectivity among all pairs of metabolites in the metabolic network for the study of the global network structure. Wrong pathways from GBA eventually lead to problematic global structures which are correct in general but wrong at the level of individual metabolites/enzymes. For example, the bow-tie structure that describes the overall mass flow in a metabolic network [5] is generally true, but the classification of metabolites into different subsets of the bow-tie structure may not be right. This may explain why these system-level organizational structures have little success in addressing real biological problems. It would therefore be of great interest to combine the two complementary approaches, using an FBA method for pathway calculation to make sure that all pathways are biologically feasible, and at the same time extend the pathway calculation to all pairs of metabolites to gain a complete picture of the connectivity at a systemic level. Establishing such a combined approach was the aim of this study. In particular, we focused on the bow-tie structure discovered previously by GBA, where nodes in a network are classified into four subsets, including the giant strongly connected component (GSC), input(IN), output(OUT) and isolated subset (IS) [5]. GSC, where all metabolites can be converted into each other through a pathway, is the core component controlling the mass flow in the metabolic network. The metabolites which can only be consumed to produce metabolites in the GSC form the IN and metabolites producible but not consumable from GSC form the OUT. All other metabolites not connected with GSC form the IS. We compared the obtained bow-tie structures with those obtained using GBA and revealed the improper classification of many metabolites by GBA. The biologically more relevant global connectivity structure obtained in this study established a crucial step for further in-depth studies on how such global structural features shape the biological functions of an organism.

## Results and discussion

### Comprehensive pathway analysis of the *E. coli* metabolic network

#### Adding Special demand reactions for metabolites with special chemical groups

**Fig. 1:**
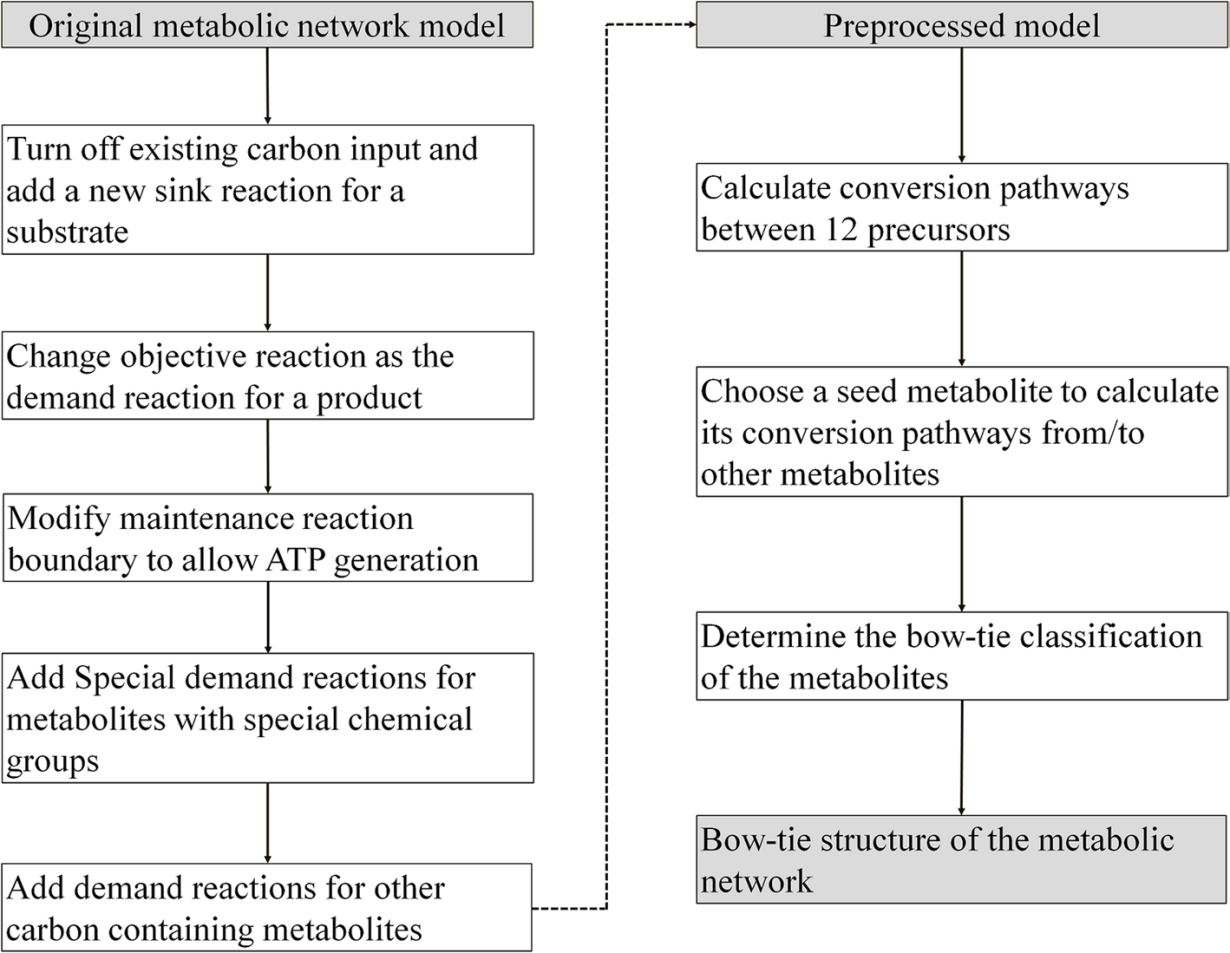
The analysis workflow for the model preprocessing and bow-tie structure calculation

The *E. coli* metabolic network model iML1515 [21] from the BiGG database [22] was first studied to examine the results and refine the pathway calculation method. We first calculated the conversion pathways between 12 biosynthetic precursors in the central pathways by switching the substrate input reaction and the objective reaction as shown in the workflow in Fig. 1 (described in detail in the methods section). Surprisingly, no pathway from acetyl-CoA and succinyl-CoA to other precursors was obtained. The reason is that the Coenzyme A group containing 21 carbon atoms in these two metabolites cannot be utilized to produce other precursors and thus cannot be balanced. However, CoA actually functions as an acyl group carrier instead of a carbon source in itself and thus only the acyl moiety of acyl-CoA should be considered as a metabolite in pathway analysis. Therefore, the model needs to be preprocessed to obtain proper pathways for CoA containing metabolites. To address this issue, we added a demand reaction for CoA in the network so that when an acyl-CoA metabolite is used as a substrate, the CoA group can be coproduced with the target product instead of being consumed as a carbon source. After this prerecession, conversion pathways between all 12 precursors were successfully calculated. This indicates that the central metabolites are fully connected, implying that they are all in the GSC part of the *E. coli* metabolic network. However, we also noticed that the obtained production pathways for acetyl-CoA and succinyl-CoA were very complex ones, rather than the commonly recognized pathways (e.g. pyruvate to acetyl-CoA through pyruvate dehydrogenase) but very complex ones. The majority of the pathways are actually used for the production of the CoA group. Therefore, in order to obtain the pathways for the acyl moiety of acyl-CoA, the demand reaction (used as the optimization objective in FBA) for a CoA-containing metabolite was changed from “acyl-CoA ◊” to “acyl-CoA ◊ CoA” when its production pathway was calculated. This way, the CoA group can be recycled and does not need to be produced from the substrate. Similarly, we added demand reactions for other functional groups such as acyl carrier protein (ACP), THF (for transferring one carbon unit), UDP, ADP, CDP (for sugar nucleotides) etc. and modified the demand reactions for metabolites containing such groups to allow their reutilization in the pathways.

#### Metabolic conversion pathways between the biosynthetic precursors

After adding special demand reactions for CoA containing metabolites, we successfully obtained conversion pathways between any pair of precursors, implying the full connection of these 12 metabolites in the *E. coli* metabolic network. Compared to GBA, one advantage of the FBA method is that the pathway conversion efficiency (represented as pathway yield) can also be calculated as rp/rs, where rp is the rate of production and rs is the substrate consumption rate. To consistently compare pathway yields between pairs of metabolites, we used the carbon yield as an indicator for pathway conversion efficiency, expressed as the total carbon atoms produced in the objective product divided by the consumed carbon atoms from the substrate. The carbon yield of a pathway connecting two carbon-containing molecules should be between 0 and 1. Among the 132 pathways between the 12 precursors, 80 of them were revealed to have a carbon yield of 1 (100 %), indicating that all carbon atoms from the substrate are transferred to the product (see Additional file 1). The lowest carbon yield among these pathways was 62 %, which was calculated for six oxaloacetate-consuming pathways for the production of f6p, g6p, and e4p. This is mainly because of the low degree of reduction (defined as the number of equivalents of available electrons per gram atom C in a compound representing its redox level [23]) of oxaloacetate, which is only 10 (2.5 per carbon atom). By contrast, the degree of reduction of g6p is 24 (4 per carbon atom) and thus the maximal carbon yield determined by the degree of reduction balance (actually a simplified representation of elemental balance) is 62 % (2.5/4), the same as the pathway yield. Therefore, even though the yield values are low for oxaloacetate-utilizing pathways, they are already at the upper boundary imposed by elemental balance.

#### Adding demand reactions for carbon-containing molecules to ensure biologically relevant pathway calculation

**Fig. 2:**
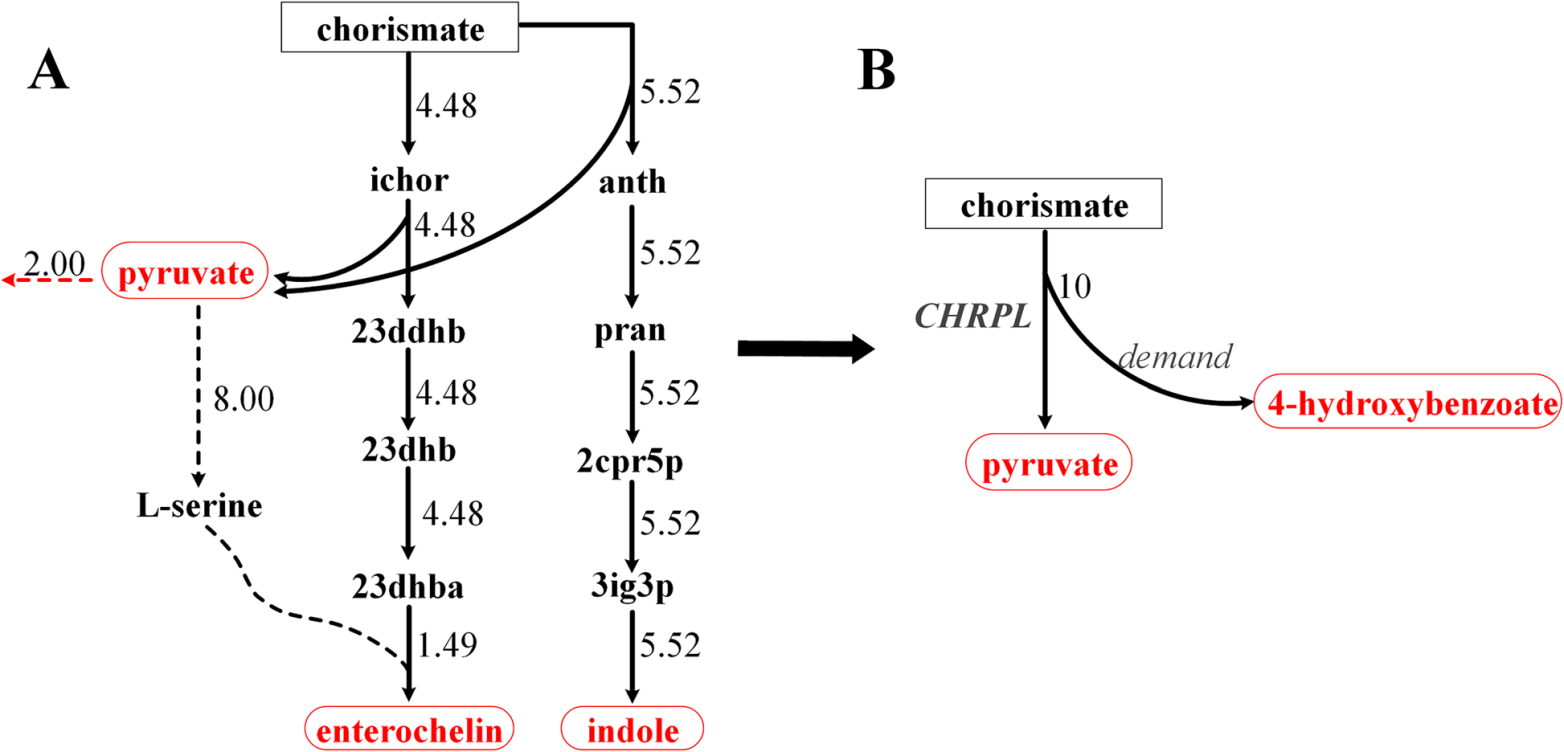
The calculated pathways from chorismate to pyruvate in the model before (**A**) and after (**B**) adding demand reactions for all carbon-containing molecules. The chorismate input flux was set at 10 mmol/gDCW/h, equal to 100 mmolC/gDCW/h as chorismate contains 10 carbon atoms. Three carbon-containing metabolites were produced in pathway A: pyruvate (3 C atoms), enterochelin (30 C) and indole (8 C). The output flux of pyruvate is only 2 mmol/gDCW/h, accounting for only 6 % of the carbon input from chorismate. The main outputs of pathway A are actually enterochelin (45 % C) and indole (44 % C). Pyruvate is just a byproduct of two reactions in the pathway, and most of the produced pyruvate is also reused through serine (dashed lines are a simplified representation of many reactions in the pathway) to produce enterochelin. Therefore, it is not reasonable to regard pathway A as converting chorismate to pyruvate. After adding demand reactions for 4-hydroxybenzoate, pathway B that contains only one main reaction in which pyruvate is a product was obtained. ichor: Isochorismate; 23ddhb: 2, 3-Dihydro-2, 3-dihydroxybenzoate; 23dhb: 2, 3-Dihydroxybenzoate; 23dhba: (2,3-Dihydroxybenzoyl)adenylate; anth: Anthranilate; pran: N-(5-Phospho-D-ribosyl)anthranilate; 2cpr5p: 1-(2-Carboxyphenylamino)-1-deoxy-D-ribulose 5-phosphate; 3ig3p: C’-(3-Indolyl)-glycerol 3-phosphate

**Fig. 3:**
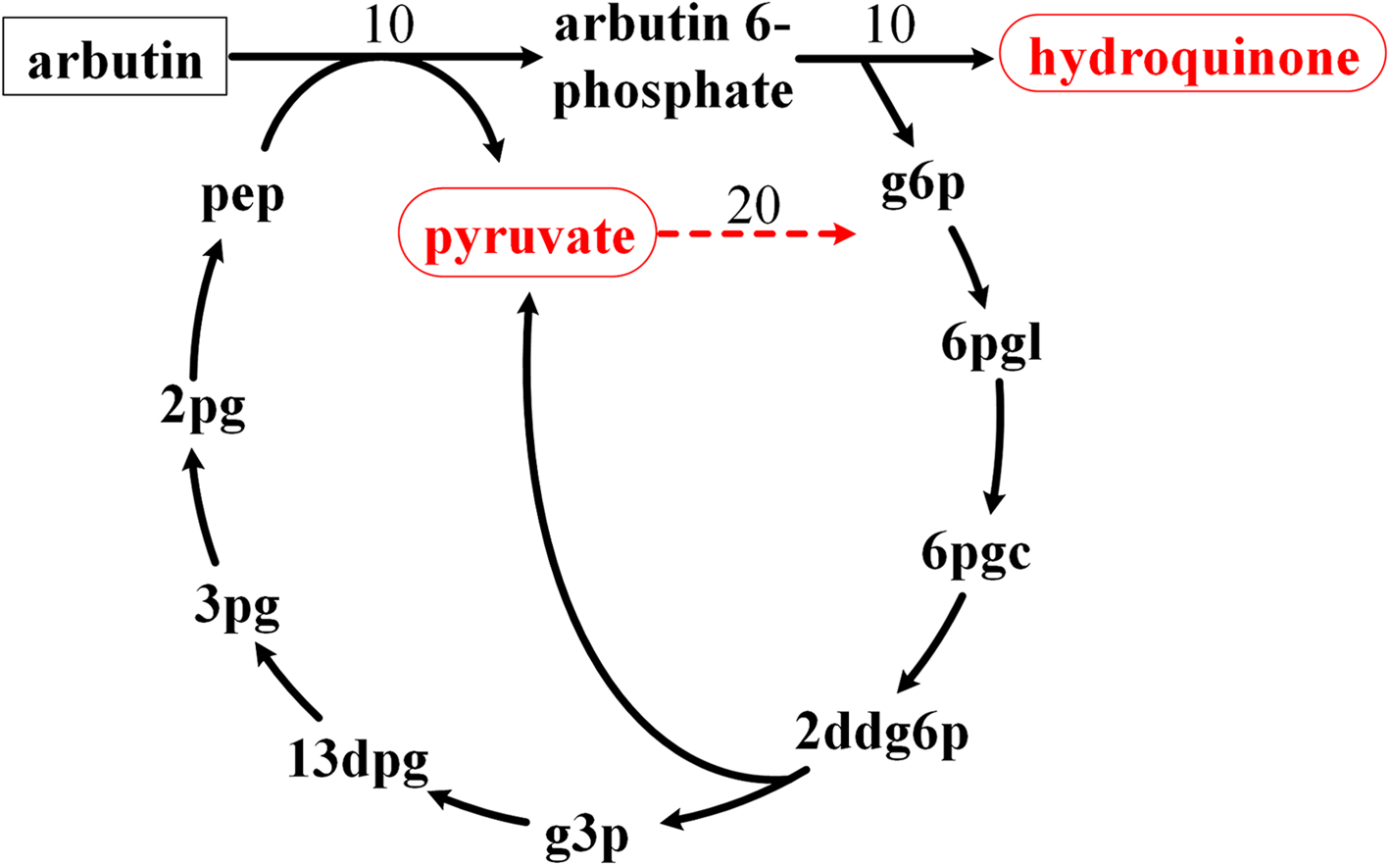
The calculated pathway from arbutin to pyruvate after adding demand reactions. Without the demand reaction for hydroquinone, no pathway from arbutin to pyruvate can be obtained as hydroquinone cannot be balanced. g6p: D-Glucose 6-phosphate; 6pgl: 6-Phospho-D-glucono-1,5-lactone; 6pgc: 6-Phospho-D-gluconate; 2ddg6p: 2-Dehydro-3-deoxy-D-gluconate 6-phosphate; g3p: Glyceraldehyde 3-phosphate; 13dpg: 3-Phospho-D-glyceroyl phosphate; 3pg: 3-Phospho-D-glycerate; 2pg: D-glycerate 2-phosphate; pep: Phosphoenolpyruvate

As all the 12 precursors are fully connected, we can choose any one of them as a precursor to calculate the associated pathways to determine its connectivity with all other metabolites (see methods section for details). With no particular reason, we chose pyruvate as the seed metabolite. Altogether 1095 metabolites were produced from pyruvate and 1072 metabolites consumed to produce pyruvate in the *E. coli* metabolic network. We noticed that for a number of metabolites the carbon yields of the pathways were very low (even less than 0.1). This implies that the target metabolite may not be the main product of the pathway. Thus, the existence of such conversion pathways is questionable. We then carefully examined the reactions in the calculated pathways. For example, the pathway from chorismate to pyruvate (carbon yield 6 %) is very complex, with over 60 reactions (Fig. 2 shows the backbone of the pathway and a list of all reactions in the pathway can be seen in additional file 3), and its main products are actually enterochelin (carbon yield 45 %) and indole (44 %). These two metabolites are produced when pyruvate is set as the target product because the iML1515 model contains corresponding exchange reactions. If we close these two exchange reactions, no pathway from chorismate to pyruvate can be calculated. However, pyruvate is actually a co-product of the CHRPL reaction (chorismate → 4-hydroxybenzoate + pyruvate), and no pathway was calculated by FBA mainly because the co-product of this reaction, 4-hydroxybenzoate, cannot be balanced by other reactions. Even though intracellular accumulation of a co-product means such pathway cannot maintain a steady state flux, most biologists may recognize that this reaction can be regarded as a biologically relevant link from chorismite to pyruvate. It would be quite controversial to exclude such pathways in the FBA based pathway analysis. To avoid such ambiguousness, instead of removing the exchange reactions, we added demand reactions for all carbon-containing molecules in the model when calculating the pathways, so that if a co-product is produced in a reaction, it can be balanced by its demand reaction. It should be noted that the addition of these new demand reactions does not change the connectivity of original network as all involved reactions in a calculated pathway between two metabolites (e.g. CHRPL reaction for chorismate to pyruvate conversion) are in the original network. The new demand reactions are only used to balance the coproducts in order to obtain steady state pathways by FBA. With this revised method for FBA based pathway calculation, we indeed obtained a simple pathway using the CHRPL reaction, with a carbon yield for pyruvate of 30 %. The addition of the demand reactions also enabled us to correctly obtain more pathways, with 1097 metabolites being produced and 1132 metabolites consumed (vs. 1095 and 1072, respectively). For example, no pathway from arbutin to pyruvate was obtained without newly added demand reactions due to the fact that one of its degradation co-products, hydroquinone, cannot be balanced by converting it to other metabolites. After adding a demand reaction for hydroquinone, a pathway from arbutin to pyruvate was obtained, with a carbon yield of 50 % (Fig. 3). A complete list of metabolites which can be produced from pyruvate or consumed to produce pyruvate and the carbon yields of the conversion pathways was presented in additional file 2.

**Fig. 4:**
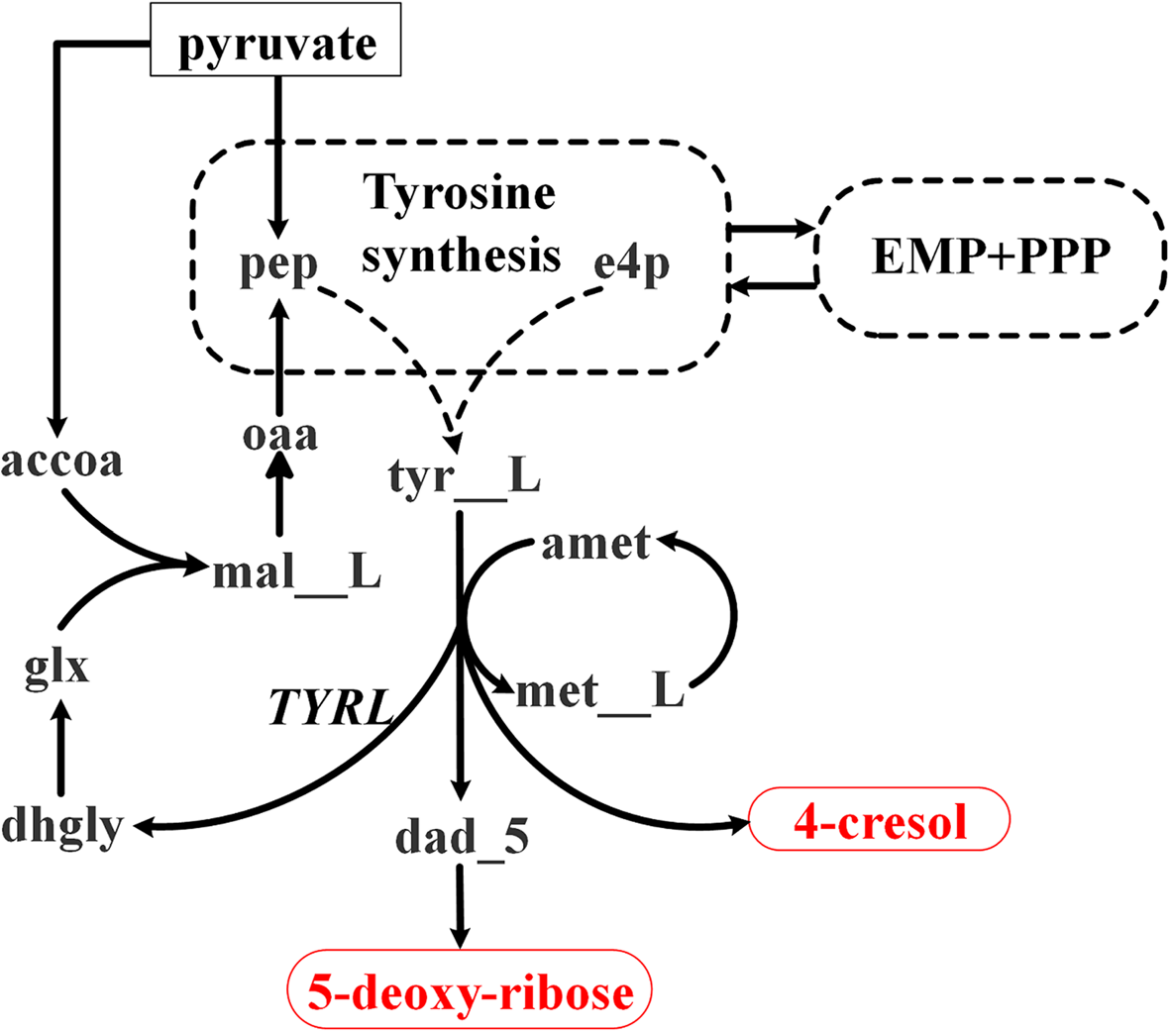
The calculated pathway from pyruvate to 5-deoxy-ribose. oaa: Oxaloacetate; pyr: Pyruvate; pep: Phosphoenolpyruvate; e4p: D-Erythrose 4-phosphate; accoa: Acetyl-CoA; mal__L: L-Malate; tyr__L: L-Tyrosine; amet: S-Adenosyl-L-methionine; met__L: L-Methionine; glx: Glyoxylate; dhgly: Dehydroglycine; dad_5: 5’-Deoxyadenosine

As no net utilization of CO_2_ was allowed in the model, the maximal carbon yield of a pathway is 1. Among the 2,229 pathways to/from pyruvate obtained for the iML1515 model, 888 pathways (174 from pyruvate and 714 toward it) did reach the maximal carbon yield, meaning that all the carbon atoms in the substrate are transferred to the product through the pathway, i.e. without carbon loss. More than 90 % (2,100/2,229) of the pathways had carbon yield values above 50 %, implying that the target products are the main products of the pathways. By contrast, the majority of the pathways with a carbon yield of less than 50 % had two main products. For example, the carbon yield from pyruvate to 5-deoxy-ribose is only 30 %. By checking the calculated pathway, we found another product in the pathway 4-cresol, with a high carbon yield of 42 % (Fig. 4). However, 4-cresol appears in only one reaction (TYRL, Tyrosine lyase) in iML1515:

$$\mathrm S-\mathrm{adenosyl}-\mathrm{methionine}\;+\;\mathrm{NADPH}\;+\;\mathrm{tyrosine}\;\rightarrow\;4-\mathrm{cresol}\;+\;5'-\mathrm{deoxyadenosine}\;+\;\mathrm{dehydroglycine}\;+\;\mathrm{methionine}\;+\;\mathrm{NADP}^+$$

This is actually the only reaction that produces dehydroglycine, a precursor of thiamine biosynthesis in *E. coli *[24]. Additionally, 5-deoxy-ribose is produced by the hydrolysis of the other co-product in this reaction, 5’-deoxyadenosine. As 4-cresol cannot be balanced by other reactions, it has to be co-produced with 5-deoxy-ribose in the pathway. This result further indicates the importance of adding other demand reactions for pathway calculation. In fact, we found that the demand reaction for 4-cresol was already intentionally added into the iML1515 model to allow 4-cresol to leave the system. Otherwise, the growth rate would be zero as there is no steady-state pathway for thiamine production due to the inability to balance 4-cresol.

Even after adding the demand reactions to allow the co-production of several metabolites, there were still many pathways with a very low carbon yield. For example, in the calculated pathway from prephenate to pyruvate, the carbon yield is only 6 %. After examining the obtained pathway, we found that it was actually not a conversion pathway from prephenate to pyruvate but a CO_2_ fixation pathway through the threonine cycle [25]. In the reaction catalyzed by prephenate dehydrogenase (PPND: NAD^+^ + prephenate → 4-hydroxyphenylpyruvate + CO_2_ + NADH), 4-hydroxyphenylpyruvate is the only main product and directly released through its demand reaction. By contrast, the NADH produced in this reaction can drive the fixation of CO_2_ through the threonine cycle to produce pyruvate. Although the carbon atoms in pyruvate do come from prephenate through the released CO_2_, it is quite a stretch to call this a conversion pathway from prephenate to pyruvate. Therefore, we excluded those pathways with a carbon yield smaller than 20 % in further global connectivity analysis.

Based on the described analysis, proper processing of the many metabolites containing certain functional chemical groups such as CoA, allowing ATP generation from the maintenance reaction, adding demand reactions for carbon-containing molecules, as well as examining and excluding low-carbon-yield pathways are all necessary steps for reliable pathway calculation.

### Global connectivity analysis of the *E. coli* metabolic network

**Fig. 5:**
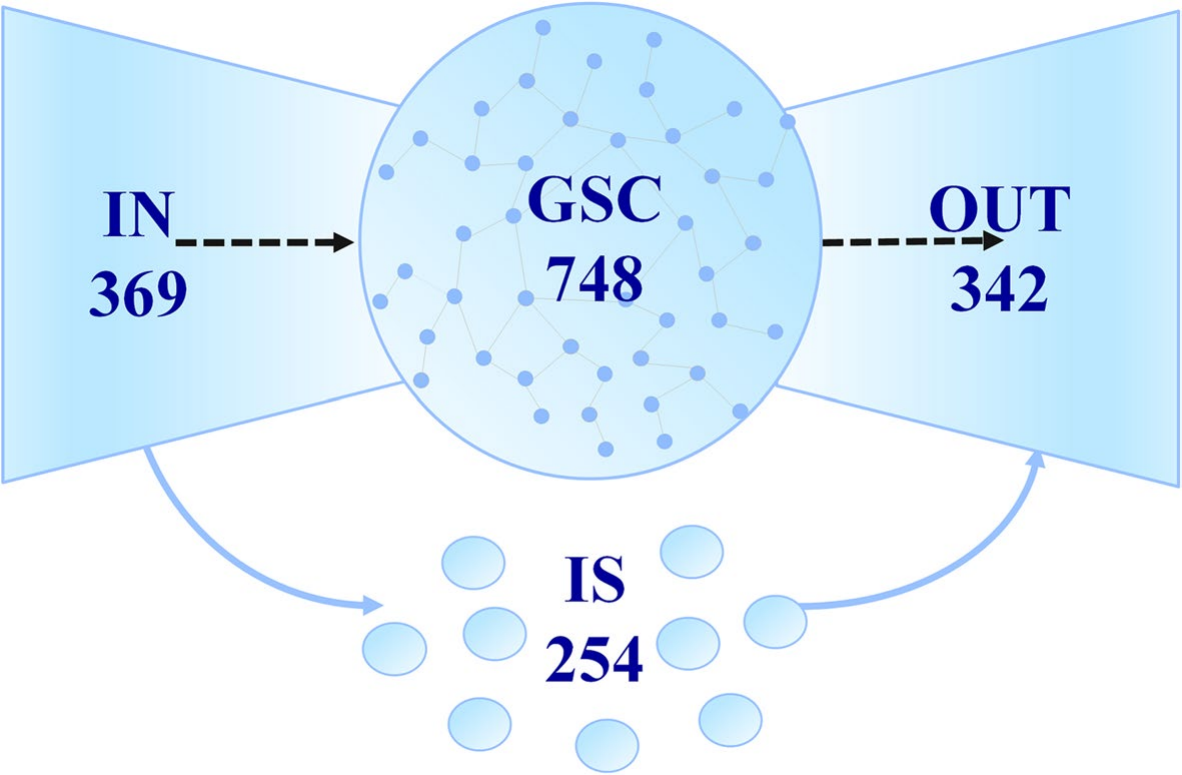
Schematic representation of the bow-tie structure of the *E. coli* metabolic network. The numbers shown are the number of metabolites in each subset of the bow-tie structure

The comprehensive FBA pathway analysis yielded over 2,000 pathways connecting 1,458 metabolites with pyruvate. Among them, 747 can be produced from pyruvate and also consumed to produce pyruvate. They can also be interconverted at least through pyruvate (although the optimal pathway with the best carbon yield may not go through pyruvate). These 748 fully connected metabolites (including pyruvate) form the giant strongly connected component (GSC) of the network. The GSC is the central part of the metabolic network and most metabolic conversions occur in the GSC. The 369 metabolites which can only be consumed to produce pyruvate (and all the metabolites in the GSC) form the IN of the bow-tie structure. Correspondingly, the 342 metabolites that can only be produced from pyruvate form the OUT. The remaining 254 of the total 1713 carbon-containing metabolites are not connected with the main part of the metabolic network and form the IS. Many of these metabolites are only involved in one reaction (dead-end metabolites), and thus cannot take part in any mass-balanced pathways. This is in agreement with previous findings that many reactions are actually blocked and cannot carry nonzero steady state fluxes [26]. However, there are also a number of metabolites in the IS which can be produced from metabolites in IN (31 metabolites) or consumed to produce metabolites in OUT (55 metabolites). A general depiction of the bow-tie structure of the *E. coli* metabolic network is shown in Fig. 5 and a complete classification of these metabolites can be found in additional file 4.

### Comparison with the bow-tie structures revealed by GBA and FBA

As the bow-tie structure has been studied by GBA, it was interesting to compare the structures obtained for the same network using the two different methods. For a like-to-like comparison, we also only considered the carbon-containing metabolites as nodes in the metabolite graph. One important issue in graph analysis of the metabolite network is the removal of links through currency metabolites [3]. As discussed previously, currency metabolites cannot by defined per se by metabolites alone, but should be defined based on their function in a reaction. For example, ATP is a currency metabolite when used for transferring phosphate together with ADP, but should be regarded as a main metabolite in the nucleotide synthesis pathway. Currency metabolites are normally used as carriers for certain functional groups, and thus often appear on two sides of a reaction as a pair. Based on this feature, instead of simple removal of currency metabolites from a network graph, we used an approach based on ranked metabolite-pairs (described in detail in the methods section) to remove most currency metabolite pairs and at the same time ensure that at least one metabolite link was kept for each reaction. A complete list of such pairs can be seen in additional file 5. As many reactions in a metabolic network are irreversible, a metabolic network should be represented as a directed graph where a reversible link is split into two directed links. Overall, we obtained a graph with 3,526 links between 1,713 metabolites for the iML1515 model. It should be noted that two or more reactions may have the same metabolite link. For example, the last reaction of valine synthesis can be catalyzed by two different enzymes: IlvE (glutamate + 2-ketovaline <=> valine + 2-ketoglutarate) and AvtA (alanine + 2-ketovaline <=> valine + pyruvate). These two reactions respectively use glutamate and alanine as amino group donors, but the main metabolite link is the same. After removing the repeat links, the number of links was reduced to 3,189.

**Fig. 6:**
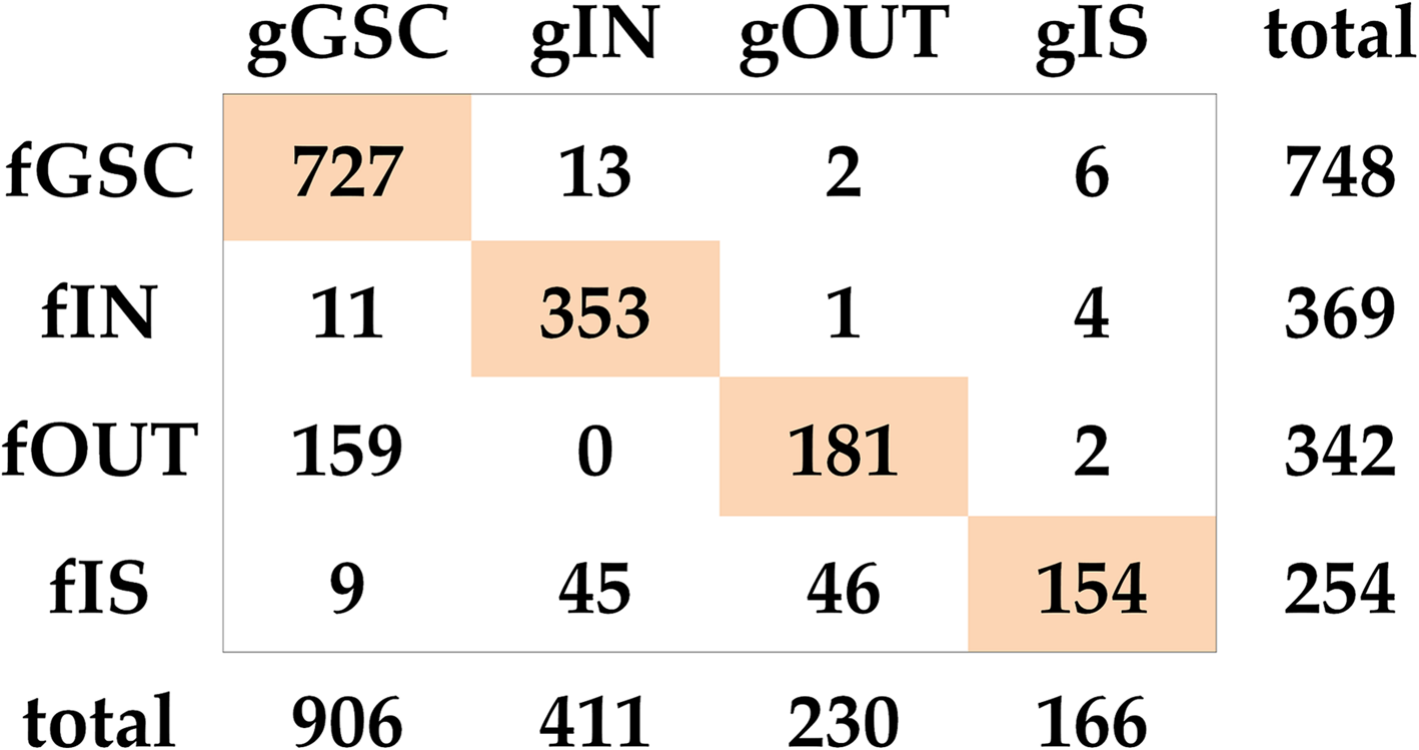
Confusion matrix comparison of the bow-tie structures from the two different methods. The subsets from GBA are prefixed with “g” and those from FBA are prefixed with “f”

**Fig. 7:**
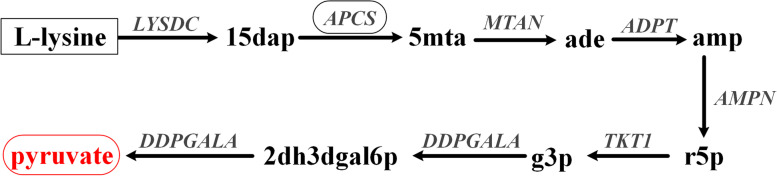
The wrong pathway from lysine to pyruvate calculated from GBA. The link from 15dap to 5mta via the APCS reaction is biologically irrelevant, as no carbon atoms are transferred from 15dap to 5mta. 15dap: Cadaverine; 5mta: S-methyl-5’-thioadenosine; ade: Adenine; amp: AMP; r5p: Alpha-D-Ribose 5-phosphate; g3p: Glyceraldehyde 3-phosphate; 2dh3dgal6p: 2-Dehydro-3-deoxy-D-galactonate 6-phosphate

With the generated graph, the bow-tie structure was calculated using the method mentioned in our previous study [5], and a summary of the results is shown in Fig. 6 (a full classification of the metabolites are detailed in additional file 6). Compared with the structure obtained by FBA pathway analysis, the classification for 1,415 (83 %) metabolites was the same. A notable difference is that the GSC from GBA (referred to as gGSC) was significantly larger (906 vs. 748) than that from FBA pathway analysis (referred to as fGSC), implying that many conversion pathways calculated from GBA are actually biologically impossible. Among the 179 metabolites in gGSC but not in fGSC, 159 can only be produced, 11 can only be consumed and 9 can be neither consumed nor produced. Typically, several amino acids such as valine, lysine, tyrosine and methionine cannot be utilized as the sole carbon sources by *E. coli *[24]. As an example, we illustrated the calculated wrong lysine utilization pathway by graphic analysis in Fig. 7. Lysine can be converted to cadaverine by lysine decarboxylase and there is no other reaction for further breakdown of cadaverine in iML1515. Accordingly, there is no pathway for converting lysine to pyruvate. However, cadaverine participates in reaction APCS as a co-substrate for aminopropylcadaverine synthesis in iML1515.

$$\mathrm{APCS}:\;\mathrm{cadaverine}\;+\;\mathrm S-\mathrm{adenosyl}\;3-(\mathrm{methylsulfanyl})\mathrm{propylamine}\;\rightarrow\;\mathrm{aminopropylcadaverine}\;+\;\mathrm S-\mathrm{methyl}-5'-\mathrm{thioadenosine}\;+\;\mathrm H^+$$

In fGSC, the co-product of this reaction is S-methyl-5’-thioadenosine, and it can thus be utilized to produce pyruvate. However, none of the carbon atoms in S-methyl-5’-thioadenosine are derived from cadaverine, and the pathway for lysine uptake obtained from GBA using this link is actually wrong. Scientists have developed atom mapping methods to address such problems, but those methods require detailed atom mapping information between the reactants and products of a reaction [16, 27]. Here, we show that this problem can be well solved by FBA pathway analysis.

The 21 metabolites presented in fGSC but not in gGSC are mainly related to the metabolism of ATP, NADH and NADPH. Many links through these metabolites were removed in the first stage of currency metabolites processing. It is very tricky to identify which links should be regarded as currency metabolite links. Even though complex rules were utilized in this study, it is still not possible to correctly identify all currency metabolite links. Various approaches have been proposed to generate metabolite graphs without meaningless currency metabolite links, but no commonly recognized standards exist to deal with the problem at the moment. This leads to inconsistencies in graph analysis of metabolic networks, especially when it is used for biological function analysis. By contrast, the FBA pathway analysis is more reliable and the calculated pathways can reflect the real mass flow in the network. Therefore, the true global connectivity structure is better represented by the bow-tie structure obtained from FBA pathway analysis.

### Bow tie structures of the metabolic networks of different organisms

To assess the broader applicability of our approach, 31 metabolic network models for different microorganisms were obtained from the BiGG database [22]. We first checked the elemental balance for all the reactions in the model, and found that only 9 of the 31 models did not have imbalanced reactions [28]. As imbalanced reactions can affect the pathway analysis result, we only chose 15 models with less than 5 imbalanced reactions. We manually examined and corrected the imbalanced reactions in these models. Notably, several reactions were identified as imbalanced simply because macromolecules such as tRNA were in the reaction equation. We did not make any change for these reactions as they should not be regarded as imbalanced. The key features of these 15 models (11 for prokaryotes and 4 for eukaryotes) are shown in Table 1. It can be seen that the scales of the metabolic networks are very different, varying from 628 metabolites for *Methanosarcina barkeri* str. Fusaro to 2,153 metabolites for *Pseudomonas putida* KT2440. The ATP maintenance reaction (ATP → ADP + Pi) was missing in a few models, and was added it to make these models consistent with the pathway calculation method used for iML1515.

**Fig. 8:**
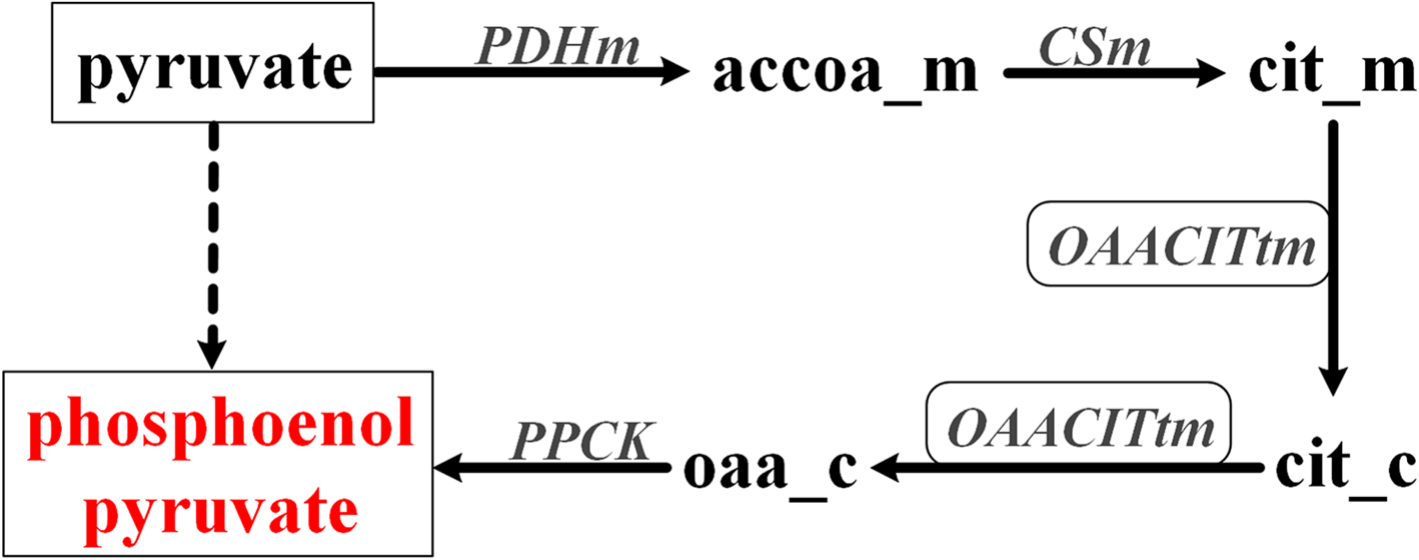
The wrong pathway from pyruvate to phosphoenolpyruvate calculated from GBA. The transport reaction OAACITtm was used twice in two different directions in this wrong pathway to link the two co-substrates of the reaction. accoa: Acetyl-CoA; cit: Citrate; oaa: Oxaloacetate

**Fig. 9:**
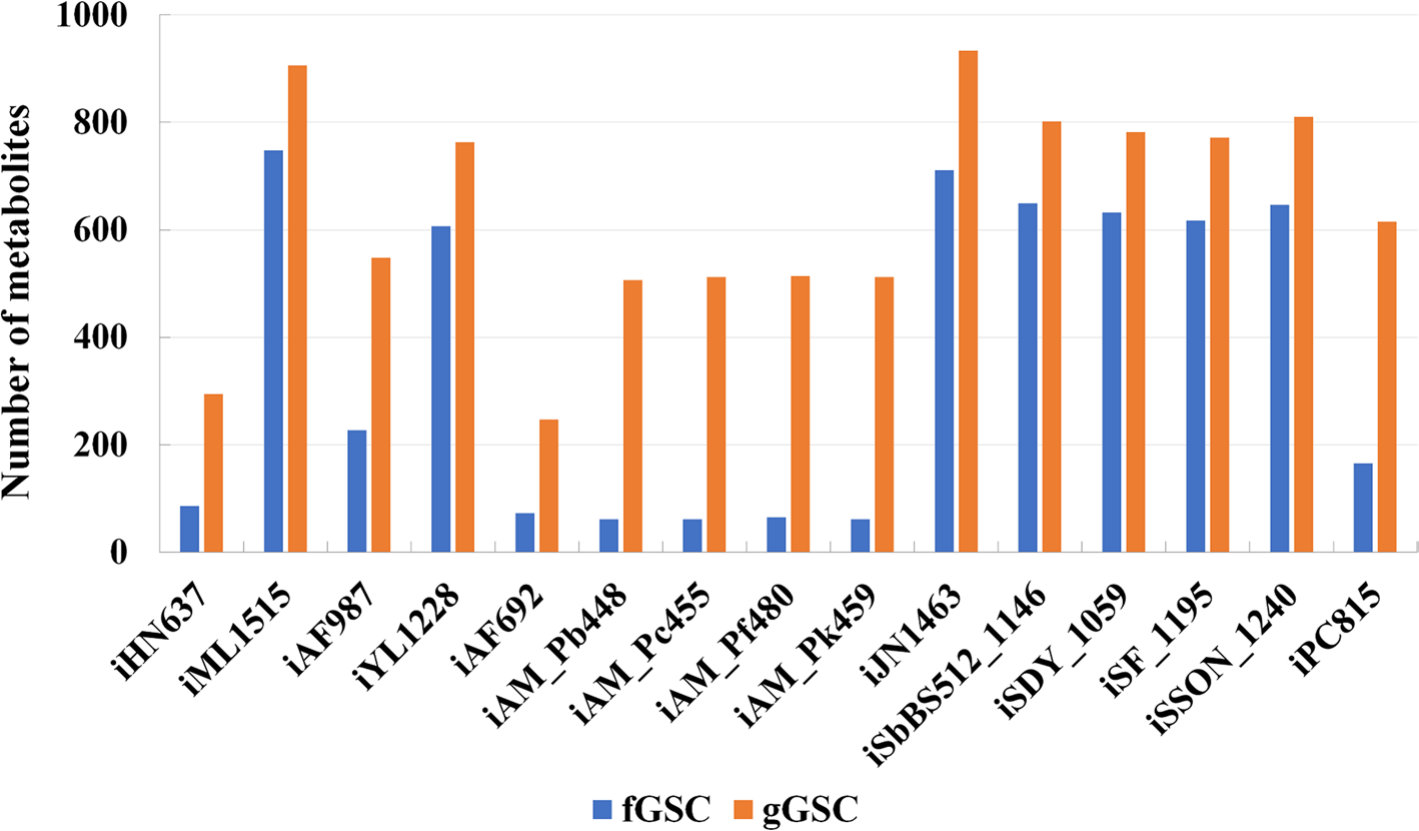
Comparison of the GSCs obtained using FBA and GBA methods for the 15 metabolic models. fGSC represents the GSC obtained using the FBA method, and gGSC represents the GSC obtained using the GBA method

**Fig. 10:**
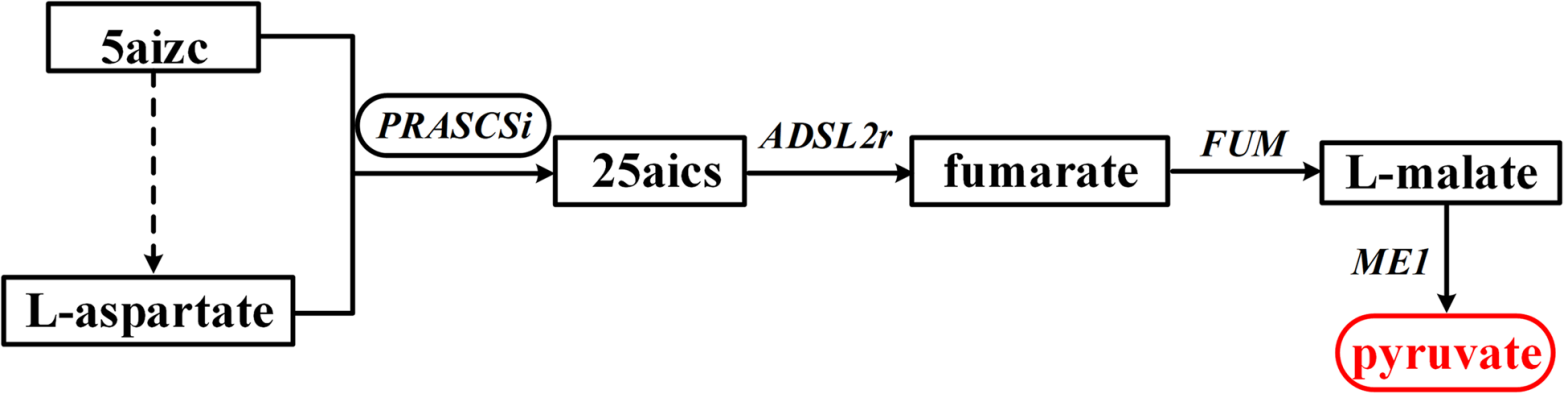
The wrong pathway from 5aizc to pyruvate calculated from GBA. Aspartate is a co-substrate for the first reaction but it cannot be produced from 5aizc. Therefore, no stoichiometrically balanced pathway from 5aizc can be obtained by FBA analysis. 5aizc: 5-amino-1-(5-phospho-D-ribosyl)imidazole-4-carboxylate; 25aics: (S)-2-[5-Amino-1-(5-phospho-D-ribosyl)imidazole-4-carboxamido]succinate

Using the same analysis workflow as that used in *E. coli* metabolic network analysis (Fig. 1), we calculated the bow-tie structures of these models and the results are summarized in Table 2 (the complete classification of the metabolites in these models can be seen in additional file 4). It can be seen that the number of metabolites in the GSC varies significantly among different organisms. Interestingly, the networks of the four eukaryotes tended to have small GSCs. This is actually in agreement with the previous findings obtained using GBA, which indicated that the metabolic networks of eukaryotes are often patchy, with long pathways between metabolites and low connectivity [3, 5]. It should also be noted that the four eukaryote models obtained from BiGG are all for species of *Plasmodium*, which are obligate parasites. This may explain the lack of many conversion pathways in these models. Table 3 shows the appearance of the central metabolites (namely in Embden-Meyerhof-Pathway (EMP), Pentose Phosphate pathway (PP), and Tricarboxylic acid cycle (TCA)) in the GSC of these models. In agreement with the common knowledge of their importance in metabolic conversion, most of these central metabolites were in the GSCs of all the studied models. The lack of the oxidative pentose phosphate pathway (6-phospho-D-gluconate and 6-phospho-D-glucono-1,5-lactone) or the inability to use acetate and succinate as substrates (succinate, acetyl-CoA and succinyl-CoA) in certain organisms can explain that some of the metabolites are not in the GSC of a few networks. Particularly, succinyl-CoA does not exist in the four eukaryote networks due to the incomplete TCA cycle of Plasmodium. D-Glucose is a common carbon source but cannot be produced from other central metabolites in the models of *Geobacter metallireducens*, *Clostridium ljungdahlii* and *Yersinia pestis* due to the lack of glucose 6-phosphatase. An unexpected result is that pyruvate is missing from the GSCs of some models. As a central metabolite connecting glycolysis and the TCA cycle, pyruvate was chosen as a seed metabolite for pathway calculation to obtain the bow-tie structure in the analysis of the *E. coli* metabolic network. However, in 3 of the eukaryote models, pyruvate can only be produced from the metabolites in the EMP and PP pathway, but cannot be utilized to produce these metabolites due to the lack of phosphoenolpyruvate synthetase (dashed line in Fig. 8). Surprisingly, pyruvate was found in the GSCs of these models when GBA was used for calculating the bow-tie structure. We examined the calculated pathways (Fig. 8), and found that the mistake was caused by the transmembrane reaction OAACITtm (cit_m + oaa_c <=> cit_c + oaa_m). This reaction was used twice in the pathway, once from cit_m to cit_c, and again from cit_c to oaa_c. Such a pathway is biologically impossible. This indicates that FBA based methods should be used to study the global connectivity structure of metabolic networks to obtain more reliable and biologically relevant results. To further illustrate this point, we also calculated the bow-tie structures for the 15 metabolic networks using GBA and compared the size of the GSCs using the two methods (Fig. 9). It can be seen that even though for some models like iML1515 the GSCs from the two methods are similar, about half of the models have significantly larger gGSCs than fGSCs. We carefully investigated the pathways linking metabolites in gGSC but not in fGSC of a model. In addition to the two pathway errors mentioned in Fig. 7 (biologically irrelevant metabolite links) and Fig. 8 (one reaction is used twice in two different directions in a pathway), another common error of the graph-based pathway is that a co-substrate in the pathway cannot be produced from the starting metabolite and thus the pathway cannot be at a steady state with non-zero fluxes. A calculated pathway from 5aizc to pyruvate from GBA is shown in Fig. 10 as an example. In this pathway, aspartate is a co-substrate for the first reaction but it cannot be produced from 5aizc. Therefore, no stoichiometrically balanced pathway from 5aizc can be obtained by FBA analysis. Furthermore, all the carbon atoms in the target metabolite pyruvate are from aspartate instead of 5aizc and this pathway is biologically irrelevant. The big differences in the calculated bow-tie structures caused by the biologically impossible pathways from GBA as shown in Fig. 9 clearly underline the importance of using FBA based pathway analysis to study the global connectivity of metabolic networks to ensure its biological usefulness.

**Table 1:** The key parameters of the 15 metabolic models used in this study

Model_ID	Organism	Domain^1^	Metabolites	Reactions	Genes
iHN637 [29]	*Clostridium ljungdahlii* DSM 13,528	p	698	785	637
iML1515 [21]	*Escherichia coli* str. K-12 substr. MG1655	p	1877	2712	1516
iAF987 [30]	*Geobacter metallireducens* GS-15	p	1109	1285	987
iYL1228 [31]	*Klebsiella pneumoniae* subsp. *pneumoniae* MGH 78,578	p	1658	2262	1229
iAF692 [32]	*Methanosarcina barkeri* str. Fusaro	p	628	690	692
iAM_Pb448 [33]	*Plasmodium berghei*	e	903	1067	448
iAM_Pc455 [33]	*Plasmodium cynomolgi* strain B	e	907	1074	455
iAM_Pf480 [33]	*Plasmodium falciparum* 3D7	e	909	1083	480
iAM_Pk459 [33]	*Plasmodium knowlesi* strain H	e	909	1079	459
iJN1463 [34]	*Pseudomonas putida* KT2440	p	2153	2927	1462
iSbBS512_1146 [35]	*Shigella boydii* CDC 3083-94	p	1910	2591	1147
iSDY_1059 [35]	*Shigella dysenteriae* Sd197	p	1888	2539	1059
iSF_1195 [35]	*Shigella flexneri* 2a str. 301	p	1917	2630	1195
iSSON_1240 [35]	*Shigella sonnei* Ss046	p	1936	2693	1240
iPC815 [36]	*Yersinia pestis* CO92	p	1552	1961	815

Notes: 1. p: prokaryote; e: eukaryote

**Table 2:** Bow-tie structure analysis of the 15 metabolic models

Model_ID	All Metabolites	GSC	IN	OUT	IS
iHN637	628	86	52	341	149
iML1515	1713	748	369	342	254
iAF987	948	227	86	479	156
iYL1228	1501	607	335	310	249
iAF692	546	73	22	303	148
iAM_Pb448	805	61	43	120	581
iAM_Pc455	808	61	49	120	578
iAM_Pf480	810	65	45	120	580
iAM_Pk459	810	61	48	120	581
iJN1463	1952	711	597	362	282
iSbBS512_1146	1730	649	248	332	501
iSDY_1059	1706	632	212	354	508
iSF_1195	1732	617	324	325	466
iSSON_1240	1751	646	340	346	419
iPC815	1395	165	339	221	670

**Table 3:** Presence of the central metabolites in the GSCs of the 15 metabolic models

Name	Pathway	# Models
3-Phospho-D-glycerate	EMP	15
D-Glucose 6-phosphate	EMP	15
D-Glycerate 2-phosphate	EMP	15
D-Glucose 1-phosphate	EMP	15
Glyceraldehyde 3-phosphate	EMP	15
3-Phospho-D-glyceroyl phosphate	EMP	15
Dihydroxyacetone phosphate	EMP	15
D-Fructose 6-phosphate	EMP	15
D-Fructose 1,6-bisphosphate	EMP	15
Phosphoenolpyruvate	EMP	15
D-Ribose 5-phosphate	PP	15
Sedoheptulose 7-phosphate	PP	15
D-Ribulose 5-phosphate	PP	15
D-Erythrose 4-phosphate	PP	15
D-Xylulose 5-phosphate	PP	15
Oxaloacetate	TCA	15
Citrate	TCA	15
Isocitrate	TCA	15
Fumarate	TCA	15
L-Malate	TCA	15
2-Oxoglutarate	TCA	14
6-Phospho-D-gluconate	PP	13
6-Phospho-D-glucono-1,5-lactone	PP	13
Succinate	TCA	13
D-Glucose	EMP	12
Pyruvate	EMP	11
Acetyl-CoA	TCA	10
Succinyl-CoA	TCA	9

## Conclusions

We present a new method to identify the bow-tie connectivity structures of metabolic networks by calculating biological pathways with FBA rather than GBA to ensure the biological feasibility of the pathways. Our study shows that certain functional chemical groups such as CoA should be processed properly and correct demand reaction should be added in order to obtain pathways representing the real mass flow, either with GBA or FBA for pathway calculation. The obtained bow-tie structure is quite different in the classification of individual metabolites. The fully connected GSCs are smaller than those from GBA due to the exclusion of many biologically impossible pathways. The combination of FBA-based pathway analysis with the study of the global connectivity structure considering pathways between all pairs of metabolites in a network offers hope for a better understanding of the impact of system level-structure on the functions of biological systems. GSC is the core part of the metabolic network where metabolic fluxes can be rerouted corresponding to different environmental conditions. In contrast fluxes in other subsets can only go in one way and there is no backward flux. This bow-tie organization principle has been used to develop a new algorithm for determination of internal fluxes using ^13^ C labeling data [37]. As there is no backward flux, metabolites in other (peripheral) subsets do not affect the ^13^ C labeling patterns of metabolites in GSC and this enables a simplified bow-tie approximation algorithm for flux determination in genome scale metabolic networks. Combination of the bow-tie structure with enzyme constrained model may also help to understand how the bow-tie structure can help an organism minimizing the enzyme switch cost in fluctuating environments (changing carbon sources) as studied recently by Basan et al [38].

## Methods

### Acquisition and quality check of the models

We downloaded the latest genome-scale metabolic models in SBML format from the BiGG Models database (http://bigg.ucsd.edu/) [22]. There are about 100 published high-quality genome-scale metabolic models for 31 microorganisms available from the BiGG database. In case that multiple models exist for the same organism, the latest model was chosen. The latest model of *Escherichia coli*, iML1515, was used as an example to illustrate the analysis methods used in this study. Element balance for all the reactions in these models was checked using the check_mass_balance function in the COBRApy package [39].

### Preprocessing the models for pathway calculation

For global connectivity analysis, we focused on the carbon flow and only calculated pathways between pairs of metabolites containing carbon atoms. These metabolites can be easily extracted based on their chemical formulas. As the original models normally use glucose as the substrate and biomass production as the objective function, we had to modify the model by updating the lower boundary of the glucose exchange reaction (EX_glc__D_e) to zero, and setting another objective function. For calculating a pathway from metabolite A to metabolite B, we added a sink reaction for A (as substrate) and set its lower boundary at -10 mmol/g/h as well as a demand reaction for B (as product) used as the optimization objective. The pFBA (Parsimonious FBA, which gives the optimal rate of the objective product but minimizes the total sum of fluxes in the pathway) function in the COBRApy package [39] was used to calculate the optimal pathway and rate for the production of B. A value of zero for the rate means that no pathway in the network can convert A to B. Multiple pathways may exist between two metabolites but for connectivity analysis we only need to consider if there are pathways from A to B rather than the number of pathways to determine the connectivity just like that used in GBA.

One problem in FBA based pathway analysis is that we often obtain more complex pathways than those from GBA. For example, the pathway from pyruvate to phosphoenolpyruvate (pep) calculated by FBA contains many reactions involved in the TCA cycle and respiratory chain for producing ATP to satisfy ATP balance. In comparison, in GBA, a simple one step pathway from pyruvate to pep can be obtained from reaction (ATP + H_2_O + pyruvate → pep + AMP + 2.0 h + Pi). This simple conversion pathway is biologically relevant as all carbon molecules from pyruvate are transferred to pep. For structure analysis, we should focus on the carbon flow and the reactions for energy balance can be ignored. To this end we added a reversible energy balance reaction (ADP + Pi <=> ATP) so that ATP can be generated from this reaction instead of a large set of reactions for ATP production from the substrate. This modification enables us to obtain the simple pathway from pyruvate to pep same as that from GBA. This made it easy for us to focus on pathways representing the actually carbon flow rather than energy production, and the calculated pathways were biologically more understandable than the complex pathways with many reactions for energy balance.

Special demand reactions were added for metabolites with special chemical groups (CoA, ACP, THF, UDP, ADP, CDP etc.) to ensure that the calculated pathways are for the production of the converted carbon-containing parts of the metabolites rather than the large carrying groups. For example, the demand reaction for an acyl-CoA metabolite was added as “acyl-CoA → CoA” so that the CoA group can be reutilized and thus not need to be synthesized from the substrate. The Sink reactions for such metabolites were updated in a similar way so that the carbon atoms in the special group of the substrate did not need to be consumed. In addition to the demand reaction for the target metabolite, we also added demand reactions for all carbon-containing molecules so that the byproducts in a reaction can be balanced in addition to the objective metabolite. The complete workflow for model preprocessing and bow-tie structure determination is shown in Fig. 1.

### Global connectivity analysis

Many metabolic networks contain over a thousand carbon-containing metabolites, leading to over a million metabolite pairs for pathway calculation. To reduce the calculation time, we simplified the process based on the transitive property of the network. Thus, if there is a pathway from A to B and a pathway from B to C, then there is also a pathway from A to C. Based on the bow-tie connectivity structure of metabolic networks discovered by GBA, we can choose any metabolite in the core part of the metabolic network (GSC) to calculate the pathways to/from it. As all metabolites in the GSC are fully connected, the connectivity of the whole GSC can be represented by one metabolite in it. However, it is problematic that one does not know which metabolites should be in the GSC beforehand. Based on previous knowledge of biochemistry, the metabolites in central pathways are more likely to be in the GSC. Therefore we chose 12 biosynthesis precursors from the central pathways (‘e4p_c’, ‘pep_c’, ‘r5p_c’, ‘oaa_c’, ‘3pg_c’, ‘pyr_c’, ‘akg_c’, ‘accoa_c’, ‘f6p_c’, ‘g6p_c’, ‘g3p_c’, ‘succoa_c’) as starting metabolites. Conversion pathways between these metabolites were first calculated to evaluate the connectivity. If these metabolites are fully connected, we can choose any one of them as a seed metabolite for pathway calculation. If not, one metabolite in the largest connected component was chosen.

After calculating all pathways to/from a seed metabolite, we classified all other metabolites based on their connectivity with the seed metabolite. Similar to the definition of the bow-tie structure in GBA, a metabolite is in the GSC if it can be produced from the seed metabolite and can also be consumed to produce the seed metabolite. It is in the IN if it can only be consumed or OUT if it can only be produced. All other metabolites are in the IS.

### Graph representation and analysis of the metabolic networks

To compare the connectivity structure revealed by FBA-based pathway analysis with that from GBA, we converted the metabolic models in SBML format into directed graphs, in which nodes represent metabolites and links represent reactions. Only carbon-containing metabolites were considered. We used a ranked metabolite pair based approach to remove most currency metabolite pairs. These pairs often appear on two sides of a reaction for transferring a chemical group such as hydride (e.g. NADH/NAD), phosphate (e.g. ATP/ADP), amine (e.g. glutamate/ketoglutarate), one carbon unit (e.g. METHF/THF), acetyl (Acetyl-CoA/acetate) etc. A complete list of such pairs can be seen in additional file 5. These pairs of metabolites were processed in a ranked order for a reaction until all the currency metabolite pairs are removed, or only one connected pair of metabolites remained. For example, in the reaction UDP + ATP = UTP + ADP, the ATP/ADP pair was removed first and the link between UDP and UTP was then kept as the main conversion of this reaction. This way, we ensure that there is at least one link for each reaction with carbon-containing molecules (excluding CO_2_, HCO_3_ and ACP).

The python-based network analysis package NetworkX (https://networkx.org/) was used to calculate the bow-tie structure using methods based on graph theory. We first calculated the strongly connected components in the network to identify the GSC. Then, we chose a node in GSC to calculate its output domain (O) and input domain (I). The IN includes nodes in I but not in the GSC, and the OUT contains nodes in O but not in the GSC. All other nodes are in the IS.

## Supplementary Information

**Additional file 1.** Pathways calculated by FBA between 12 precursors in iML1515.

**Additional file 2.** Pathways between pyruvate (the seed metabolite) and other metabolites in iML1515

**Additional file 3.** The calculated pathway from chorismate to pyruvate before adding demand reactions for all carbon-containing metabolites.

**Additional file 4.** Classification of metabolites in the bow-tie structures of the models.

**Additional file 5.** List of currency metabolites and metabolite pairs.

**Additional file 6.** Comparison of the bow-tie structures obtained using GBA and FBA methods.

## Data Availability

All the codes and required data for the analysis are available from https://github.com/tibbdc/bow-tie.

## Abbreviations

GSMNs: Genome-scale metabolic networks
FBA: Flux balance analysis
GBA: Graph based analysis
GSC: giant strongly connected component
IN: input subset
OUT: output subset
IS: isolated subset
CHRPL: chorismate pyruvate lyase
TYRL: tyrosine lyase
PPND: prephenate dehydrogenase
APCS: aminopropylcadaverine synthase
EMP: Embden-Meyerhof-Pathway
PP: Pentose Phosphate pathway.
TCA: Tricarboxylic acid cycle
